# Autophagy enhances the replication of Peste des petits ruminants virus and inhibits caspase-dependent apoptosis in vitro

**DOI:** 10.1080/21505594.2018.1496776

**Published:** 2018-08-01

**Authors:** Bo Yang, Qinghong Xue, Xuefeng Qi, Xueping Wang, Peilong Jia, Shuying Chen, Ting Wang, Tianxia Xue, Jingyu Wang

**Affiliations:** a College of Veterinary Medicine, Northwest A&F University, Yangling, China; b China Institute of Veterinary Drug Control, Beijing, China

**Keywords:** Peste des petits ruminants virus (PPRV), autophagy, apoptosis, replication, caprine endometrial epithelial cells (EECs)

## Abstract

Peste des petits ruminants (PPR) is an acute and highly contagious disease in small ruminants that causes significant economic losses in developing countries. An increasing number of studies have demonstrated that both autophagy and apoptosis are important cellular mechanisms for maintaining homeostasis, and they participate in the host response to pathogens. However, the crosstalk between apoptosis and autophagy in host cells during PPRV infection has not been clarified. In this study, autophagy was induced upon virus infection in caprine endometrial epithelial cells (EECs), as determined by the appearance of double- and single-membrane autophagy-like vesicles, LC3-I/LC3-II conversion, and p62 degradation. We also found that PPRV infection triggered a complete autophagic response, most likely mediated by the non-structural protein C and nucleoprotein N. Moreover, our results suggest that autophagy not only promotes the replication of PPRV in EECs but also provides a potential mechanism for inhibiting PPRV-induced apoptosis. Inhibiting autophagosome formation by wortmannin and knocking down the essential autophagic proteins Beclin-1 and ATG7 induces caspase-dependent apoptosis in EECs in PPRV infection. However, inhibiting autophagosome and lysosome fusion by NH_4_Cl and chloroquine did not increase the number of apoptotic cells. Collectively, these data are the first to indicate that PPRV-induced autophagy inhibits caspase-dependent apoptosis and thus contributes to the enhancement of viral replication and maturity in host cells.

## Introduction

Peste des petits ruminants virus (PPRV), the aetiological agent of peste des petits ruminants (PPR), belongs to the genus *Morbillivirus* in the family *Paramyxoviridae* [1]. PPR is an acute, highly contagious, world organization for animal health (OIE) notifiable and economically important transboundary viral disease of sheep and goats that is associated with high morbidity and mortality. Clinically, the disease is characterized by a high fever, conjunctivitis, oculo-nasal discharge, necrotizing and erosive stomatitis, and diarrhoea [2]; notably, PPRV infection often causes foetal mummification, abortions late in pregnancy, and the birth of dead lambs or weak lambs that die within a couple of days [3,4]. The PPRV genome of approximately 16 kilobases (kb) encodes six structural proteins, namely, the nucleocapsid (N), phospho (P), matrix (M), fusion (F), haemagglutinin (H) and large (L) proteins in the 3′ to 5′ direction (3′-N-P-M-F-H-L-5′); two non-structural proteins (C/V) are also encoded due to the RNA editing of the phosphoprotein gene. Four different lineages of PPRV (I, II, III, and IV) have been defined worldwide based on the molecular epidemiology of the “N” and “F” gene sequences of the virus [5,6]. PPRV is currently endemic in most of Africa, the Middle East, South Asia and China and causes significant economic losses [2,7].

Cell death in multicellular organisms is classified as autophagy, apoptosis, and necrosis, each of which are morphologically distinct [8]. Autophagy is an evolutionarily conserved intracellular process that involves the formation of a double-membrane structure called the autophagosome. It delivers misfolded or long-lived cytoplasmic proteins and damaged organelles to lysosomes for degradation and recycling [9,10]. Currently, LC3 is widely used as a marker for monitoring autophagy [11,12]. In addition to LC3, the polyubiquitin-binding protein sequestosome 1 (SQSTM1, also called p62), whose degradation is increased during autophagy, is also frequently used to assist in assessing autophagic flux [12,13]. Autophagy also contributes to innate and adaptive immunity against a wide variety of intracellular microbial pathogens, including bacteria, viruses and protozoa [14,15]. An increasing amount of evidences suggest that the autophagy processes are also exploited by many viruses for their replication, such as the measles virus (MeV) [16], PPRV [17], hepatitis C virus [18], classical swine fever virus (CSFV) [19], porcine reproductive and respiratory syndrome virus (PRRSV) [20], avian reovirus [21], and influenza A virus [22]. However, it has been demonstrated that autophagy is involved in the elimination of the herpes simplex virus [23], foot-and-mouth disease virus [24], human rotavirus [25] and cytomegalovirus [26]. Thus, there is an extremely complex interaction between autophagy and invading viruses. In contrast, apoptosis is regulated by CASPs/caspases, which are apoptosis-related cysteine peptidases [27,28]. Two main signals induce apoptosis, namely, the intrinsic and extrinsic pathways. The induction of the intrinsic pathway results in mitochondrial outer membrane permeabilization, thus triggering CASP3/caspase-3 by activating CASP9/caspase-9 [27]. The extrinsic pathway activates CASP3 via CASP8/caspase-8 cleavage in a death receptor-mediated manner [27]. Apoptosis could also be considered a defence mechanism against virus replication because it triggers cell death [29]. Moreover, there are a variety of mechanisms by which the apoptotic and autophagic pathways are intertwined to affect cell fate [30]. It has been demonstrated that viruses can exploit autophagy and apoptosis for their replication [20,31,32]. A previous study suggested that autophagy can promote PRRSV replication by postponing apoptosis through the formation of a Bad-Beclin-1 complex [33]. Autophagy is involved in the apoptosis and death of T lymphocytes in the spleen of pigs infected with CSFV [34]. Moreover, autophagy triggered by genotype VII Newcastle disease virus infection was essential for viral replication, NDV-induced apoptosis, and cell survival in chicken cells and tissues [35].

It has been demonstrated that PPRV infection is closely related to apoptosis and autophagy [17,36,37]. However, the functional interaction between autophagy and apoptosis during PPRV infection in caprine-derived cells is largely unknown. In this study, we first investigate the roles of apoptosis and autophagy in PPRV-infected caprine EECs and then reveal a novel regulatory mechanism by which autophagy limits apoptosis and contributes to virus infection.

## Materials and methods

### Cell line and virus

Caprine endometrial epithelial cells (EECs) were immortalized by transfection with human telomerase reverse transcriptase (hTERT) to maintain the secretory function of the primary cells as previously described [38,39]; these cells were kindly provided by Prof Yaping Jin (Northwest A&F University Yangling, Shaanxi, China). The cells were cultured in Dulbecco’s minimal essential medium/F-12 Ham’s medium (DMEM/F12) supplemented with 10% foetal bovine serum (FBS, Gibco), 100 IU/ml penicillin, and 10 μg/ml streptomycin at 37°C in 5% CO_2_.

The PPRV vaccine strain Nigeria 75/1 was from our laboratory culture collection. The viral stock was prepared by collecting the infected cell supernatant when a cytopathic effect (CPE) was apparent in approximately 80% of the cells. To determine the virus titres, cells cultivated in 96-well plates were inoculated with 10-fold serial dilutions of the virus and incubated at 37°C for 5–7 d. The viral titres were estimated with the Reed and Muench method and expressed as the 50% tissue culture infective dose (TCID_50_)/ml. The multiplicity of infection (MOI) was confirmed according to the virus titre of the respective cell line. The UV inactivation of PPRV was performed by irradiation with UV light for 30 min at room temperature. The infectivity of UV-treated PPRV was confirmed by detecting the virus titres as described above, and the MOIs were the same as those for the untreated viruses.

### Antibodies, chemicals and other reagents

An anti-PPRV-N monoclonal antibody was provided by the China Animal Health and Epidemiology Center (Qingdao, China). Anti-LAMP1 (ab25630), anti-p62/SQSTM1 (ab101266), anti-caspase-9 (ab69514) antibodies were purchased from Abcam (Cambridge, MA). Anti-ATG7 (8558), anti-Beclin-1 (3459), anti-caspase-3 (9665) and anti-caspase-8 (9746) primary antibodies were obtained from Cell Signaling Technology (Boston, MA). An anti-β-actin (HC201) primary antibody and PE-conjugated goat anti-rabbit (HS121) secondary antibodies were purchased from TransGen Biotech (Beijing, China). A rabbit polyclonal anti-LC3B (L7543) primary antibody and horseradish peroxidase-conjugated goat anti-mouse (A9917) and anti-rabbit (A0545) secondary antibodies, as well as fluorescein isothiocyanate (FITC)-conjugated goat anti-rabbit (F9887) and tetramethyl rhodamine isothiocyanate (TRITC)-conjugated goat anti-mouse (T7782) secondary antibodies, were all purchased from Sigma-Aldrich (St. Louis, MO). Additionally, rapamycin (R0395), NH_4_Cl (V900222), chloroquine (C6628), protease inhibitor E64d (E8640) and Hoechst 33342 (B2261) were purchased from Sigma-Aldrich (St. Louis, MO). Wortmannin was purchased from Gene Operation (Ann Arbor, MI). ShRNAs targeting autophagy-related gene 7 (ATG7) and Beclin-1 and a scrambled shRNA were kindly provided by Dr. Hung-Jen Liu (Institute of Molecular Biology, National Chung Hsing University, Taichung, Taiwan).

To construct pCDNA3.1-HA-GST, pCDNA3.1-HA-N, pCDNA3.1-HA-F, pCDNA3.1-HA-C, pCDNA3.1-HA-H and pCDNA3.1-HA-V, the gene sequences for PPRV N, F, C, H, V and GST were amplified by RT-PCR and subcloned into pCDNA3.1(+) vectors. All of the constructs were confirmed by DNA sequencing.

### Virus infection and cell treatment

According to the requirements of different experiments, EECs were infected with PPRV at an MOI of 1 or mock-infected with phosphate-buffered saline (PBS). After 1 h incubation at 37°C, the unbound viruses were removed by washing thrice with PBS; then, the EECs were cultured in DMEM/F12 supplemented with 2% FBS at 37°C for the indicated time points. For the autophagy induction and inhibition experiments, EECs were pre-treated with rapamycin (100 nM), NH_4_Cl (5 mM), chloroquine (10 µM) and wortmannin (200 nM) for 6 h prior to viral infection. Viral adsorption was performed at 37°C for 1 h. The cells were then incubated in fresh medium containing rapamycin (100 nM), NH_4_Cl (5 mM), chloroquine (10 µM) and wortmannin (200 nM) until the harvesting of the cells or the culture medium. Moreover, the same amount of dimethyl sulfoxide (DMSO) was added to the control group. For the analysis of autophagic flux, EECs with identical growth status were divided into three groups: (a) Mock-infected EECs (mock). In this group, EECs were cultured in DMEM/F12 supplemented with 10% FBS for 24 h and then cultured in 2% DMEM in the absence or presence of the protease inhibitor E64d (10 µg/ml) for 48 and 72 h. (b) Rapamycin-pre-treated EECs (rapamycin). In this group, EECs were cultured in 10% DMEM/F12 for 24 h and treated with 100 nM rapamycin for 12 h, after which the rapamycin was removed. Subsequently, the cells were mock-infected with PBS and then cultured in 2% DMEM in the absence or presence of E64d for 48 and 72 h. (c) PPRV-infected cells (PPRV). In this group, EECs were cultured in 10% DMEM for 24 h, infected with PPRV and then cultured in 2% DMEM in the absence or presence of E64d for 48 and 72 h.

### Transmission electron microscopy (TEM)

Ultra-thin sections (70 nm) of cells were prepared and examined under a Hitachi HT-7700 transmission electron microscope (Hitachi High Technologies Co., Japan) as described previously [40]. Briefly, EECs were mock-infected or infected with PPRV Nigeria 75/1 at an MOI of 1 for 48 h. The cells were then washed three times with PBS and collected by centrifugation at 800 × *g* for 5 min; one drop of 2% preheated agarose was added to the cell pellet and uniformly mixed. After solidification, the agar was cut into 1-mm^3^ blocks and put in a 2.5% glutaraldehyde/0.1 M phosphate buffer solution at 4°C for fixation; the sections were washed 3 times with phosphate buffer solution for 10 min and post-fixed in 1% osmium tetroxide at 4°C for 1 h. Following dehydration with a graded series of ethanol solutions, the cells were embedded in a mixture of Epoxy 812 and warmed at 35°C, 45°C, and 60°C for 12 h, 12 h, and 48 h, respectively. Ultra-thin sections were prepared and stained with 4% uranyl acetate and observed under a transmission electron microscope.

### Immunoblotting analysis

EECs were intentionally infected with the Nigeria 75/1 strain of PPRV. Protein samples were prepared from harvested cells at increasing time intervals following infection and were subjected to immunoblotting using primary antibodies that recognize different forms of the cellular proteins. One monoclonal antibody (MAb) that specifically recognizes the PPRV N protein was used to track the progression of infection. At the indicated time points, cell lysates were generated by adding 5× SDS-PAGE sample buffer to the collected cells. The samples were boiled for 10 min, separated by SDS-PAGE, and then transferred onto 0.22-µm polyvinylidene difluoride membranes (Millipore, Billerica, MA). The membranes were blocked with 5% non-fat milk and incubated with primary antibodies, followed by HRP-conjugated secondary antibodies. The bound antibodies were detected with ECL immunoblotting detection reagents (Millipore, Billerica, MA). Images were obtained with a CanoScan LiDE 100 scanner (Canon), and the intensity of the target protein blots was analysed by ImageJ software (NIH).

### Confocal immunofluorescence microscopy

EECs were grown on coverslips to ~ 80% confluency and infected with PPRV at an MOI of 1. At the indicated times post-infection, the cells were washed four times with PBS and fixed in 4% paraformaldehyde. The cells were washed again four times with PBS and treated with 0.1% Triton X-100 for 15 min. The cells were then incubated with 1% bovine serum albumin (BSA) and the appropriate primary antibodies for 1 h at 37°C. Then, the cells were washed and incubated simultaneously with FITC- or TRITC- conjugated secondary antibodies. Finally, the cells were treated with a Hoechst 33342 solution for 5 min and analysed under a confocal microscope (CLSM Leica SP8, Germany). In addition, 3D images were sequentially scanned and recorded using a Leica TCS SP8 laser scanning confocal microscope and analysed with Imaris software (Bitplane) for visualization, manual segmentation and surface rendering.

To further analyse the effect of viral proteins on autophagosomes, EECs were grown to approximately 80% confluence and transfected with the pCDNA3.1-HA-N, pCDNA3.1-HA-F, pCDNA3.1-HA-C, pCDNA3.1-HA-H and pCDNA3.1-HA-V plasmids by using Turbofect transfection reagent (Thermo Scientific, Rockford, IL) as described below. After 48 h, immunofluorescence and immunoblotting analyses were performed as described above. Antibodies against LC3B and HA were used to determine the localization of the target proteins in the treated cells. The HA (red) and LC3 (green) proteins were examined by confocal microscopy. Cells transfected with the pCDNA3.1-HA-GST plasmid served as the control group.

### Transfection and gene silencing with shRNA

EECs grown to 80% confluence in 6-well cell culture plates were transfected with ATG7, Beclin-1 or scrambled shRNA by using Turbofect transfection reagent (Thermo Scientific, Rockford, IL). Briefly, 4 μg/well shRNA was diluted in 200 μl of serum-free Opti-MEM medium, and 6 μl of transfection reagent was pipetted directly into the medium containing the diluted shRNA plasmids and incubated at 25°C for 20 min. The cell culture medium was removed and replaced with 2 ml of Opti-MEM containing the transfection complex and further cultured at 37°C. After 48 h, the reaction mixture was discarded, and the cells were then infected with PPRV at an MOI of 1. Following 1 h of PPRV absorption, the cells were incubated in fresh medium until the harvesting of the cells or the culture medium at the indicated times post-infection. The silencing efficiency was measured by immunoblotting analysis. Scrambled shRNA was used as a negative control.

### Quantification of viral RNA

EECs were pre-treated with a chemical autophagy inhibitor or activator and shRNAs against the ATG7 and Beclin-1 genes. Viral adsorption was performed at 37°C for 1 h. The cells were then incubated until the harvesting of the cells or the culture medium. The culture medium was collected to detect the extracellular viral RNA levels and titres. The cells were added to fresh medium and freeze-thawed three times; then, the cells were collected by centrifugation at 800 × *g* for 5 min at 4°C. The supernatants were collected to detect the intracellular viral RNA levels and titres. Real-time quantitative reverse transcriptase polymerase chain reaction (qRT-PCR) was used to detect virus copies in the same volume of medium. Total RNA was extracted from the filtered supernatant with a viral RNA extraction kit (Qiagen, Germany) and purified according to the manufacturer’s recommendations, and cDNA synthesis was performed using EasyScript First-Strand cDNA SuperMix (TransGen Biotech, Beijing, China) according to the manufacturer’s instructions. For PPRV-specific detection, a primer pair (Forward: TATAGCCACGGGGGTGAAGA and Reverse: CATCGCTGTCGTCAGATCCA) targeting the region corresponding to the P gene was used. Real-time RT-PCR was performed using TransStart Top Green qPCR SuperMix (TransGen Biotech, Beijing, China) and an iQ5 iCycler detection system (Bio-Rad). The recombinant plasmid containing the PPRV P gene was used to construct a standard curve for calculating the viral copy number in different samples.

### TUNEL staining

The apoptotic events post-infection were examined by one-step terminal deoxynucleotide transferase-mediated d-UTP biotin nick end labeling (TUNEL) assays. EECs were cultured in 12-well plates to ~ 80% confluency and infected with PPRV at an MOI of 1. At the indicated times post-infection, the cells were washed four times with PBS and fixed in 4% paraformaldehyde. The following staining procedures were performed according to the manufacturer’s instructions (Vazyme Biotech, Nanjing, China), and the cells were analysed under a confocal microscope (CLSM Leica SP8, Germany).

### Flow cytometry analysis

Flow cytometry was performed to determine apoptosis using an annexin V-FITC double-staining apoptosis detection kit (KeyGen Biotech, Nanjing, China) according to the manufacturer’s protocol. EECs were pre-treated with rapamycin, NH_4_Cl, chloroquine and wortmannin for 6 h and transfected with shRNA prior to viral infection; then, the cells were infected with PPRV at an MOI of 1. For flow cytometry analyses, the cells were harvested, washed thrice with phosphate buffer saline (PBS), centrifuged, and suspended in 500 μL of 10× binding buffer, followed by treatment with 10 μl of FITC-labelled annexin V per sample for 10 min at room temperature. Then, the infected cells were stained with 5 μl of propidium iodide (PI) per sample for 5 min, followed by analysis with a Coulter Epics XL FACS system (Beckman Coulter, Brea, CA, USA). Annexin V-positive and PI-negative cell populations in the lower right quadrant of the Annexin V versus PI FACS plots were considered apoptotic cells.

### Caspase activity detection

Caspase colorimetric assay kits (Keygen Biotech, China) were used to measure the activity levels of caspase-3, caspase-8 and caspase-9. EECs were pre-treated with rapamycin, NH_4_Cl, chloroquine and wortmannin for 6 h and transfected with shRNA prior to viral infection; then, the cells were infected with PPRV at an MOI of 1. At the indicated time points, the cells were treated with lysis buffer, and the protein concentrations were measured using BCA protein assay reagent (Vazyme, NJ, USA). Then, 150-μg lysates of each sample were loaded into microplates and incubated with each caspase substrate at 37°C for 4 h; after that, the absorbance values of the samples were measured at 405 nm in a microplate spectrophotometer (Infinite 200 PRO NanoQuant, Tecan, Switzerland).

### Statistical analysis

The data are expressed as the means ± standard deviation (SD). The significance of the variability between the different treatment groups was calculated with two-way ANOVA, followed by Tukey’s multiple comparisons test using GraphPad Prism 6.0 software (Graph Pad Software Inc., San Diego, CA, USA). A *P* value of < 0.05 was considered statistically significant.

## Results

### PPRV infection increases the levels of autophagic markers in EECs

**Figure 1.**
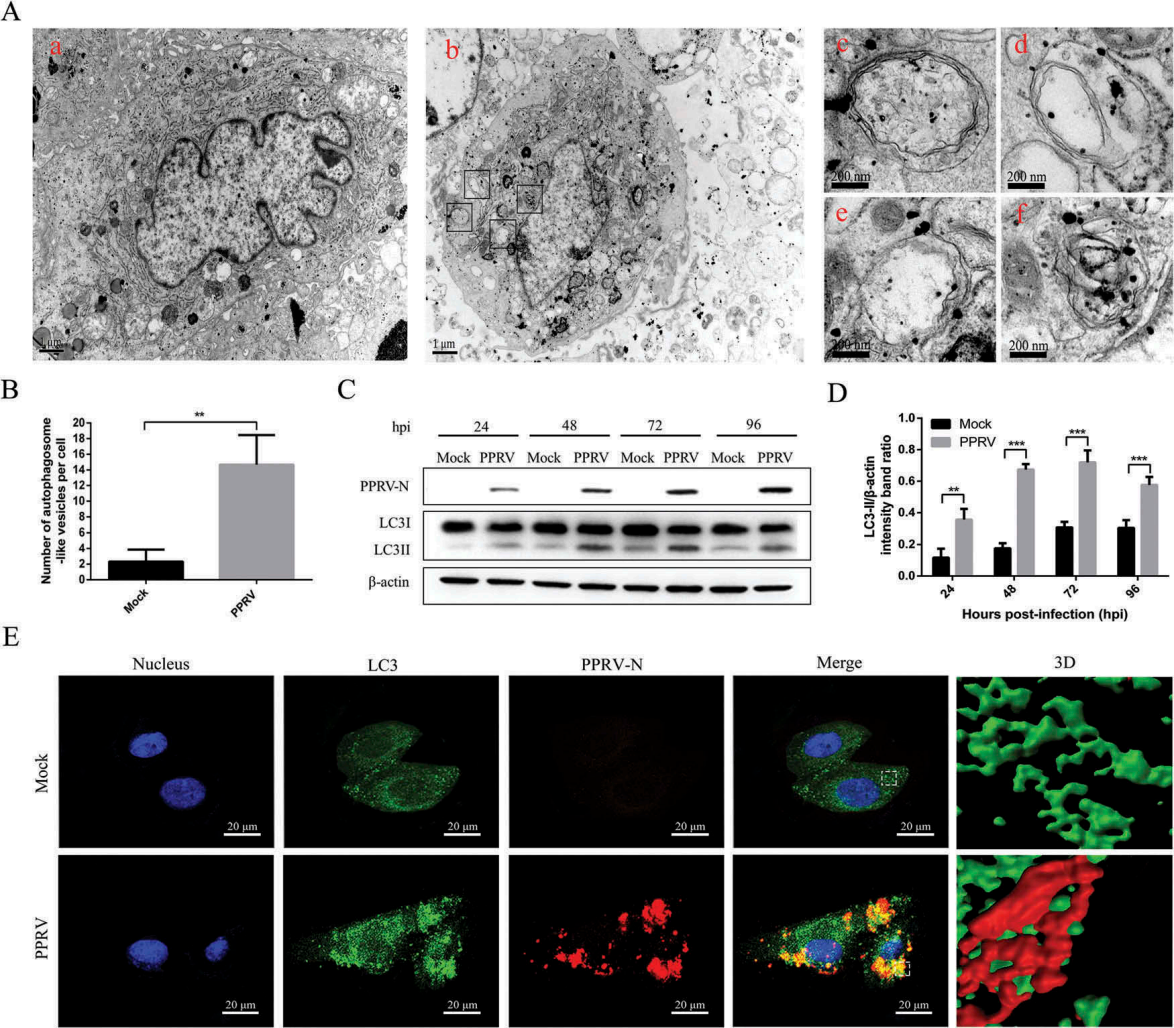
PPRV infection induces autophagy in EECs. (A) PPRV infection increases the formation of autophagosome-like vesicles. EECs were mock-infected (a) or infected with PPRV Nigeria 75/1 (b) at an MOI of 1 for 48 h. Then, the mock- and PPRV-infected cells were fixed and processed for electron microscopy analysis. (c-f) Magnified views of the autophagosome-like vesicles are enclosed by four black square frames in part b. Scale bars, 1 μm (a and b) and 200 nm (c-f). (B) Quantification of the number of autophagosome-like vesicles per cell profile in mock- and PPRV-infected cells. The average number of the vesicles in each cell was obtained from at least 10 cells per experimental condition. (C) EECs were mock-infected or infected with PPRV (MOI = 1) for 24, 48, 72 and 96 h. At the end of the infection, the expression levels of LC3, PPRV-N, and β-actin (loading control) were analysed by immunoblotting with specific antibodies. (D) The relative quantification of LC3-II protein levels compared to β-actin protein levels was determined by densitometry. (E) EECs were mock-infected or infected with PPRV (MOI = 1) for 72 h. Then, the cells were fixed and processed for indirect immunofluorescence using antibodies against LC3 and N protein. Cell nuclei were counterstained with Hoechst 33342. 3D surface rendered (SR) images in the boxed area are shown in the right panels. Scale bars, 20 μm. The data represent the mean ± SD of three independent experiments. Two-way ANOVA; **P < 0.01; ***P < 0.001.

To determine whether PPRV infection regulates cellular autophagy, transmission electron microscopy (TEM) was used to perform ultrastructural analyses of the permissive cells infected with the Nigeria 75/1 strain of PPRV. We found that the numbers of single and double autophagosome-like membrane vesicles were increased in the cytoplasm of PPRV-infected EECs (Figure 1(A), b), and many recognizable cytoplasmic contents or partially degraded organelles were sequestered in these vesicles (Figure 1(A), c-f). Similar vesicles were rarely seen in the uninfected cells (Figure 1(A), a). Quantitative analyses also showed a significant increase in the quantity of single- and double-membrane vesicles in PPRV-infected cells compared with uninfected cells (Figure 1(B)).

To further analyse if the autophagy machinery can be triggered by PPRV infection, we next examined LC3 conversion, an important hallmark of autophagy, using immunoblotting analyses. The results of the immunoblotting analyses showed that the amount of LC3-II was increased with the progression of PPRV infection (Figure 1(C)). More importantly, it was found that LC3-II levels began to increase at 24 hpi, in concert with N expression (Figure 1(C). These data indicate that the early stages of autophagy are induced upon PPRV infection in EECs. Because the amount of LC3-II correlates well with the number of autophagosomes, the ratio of LC3-II to β-actin levels in cells is currently regarded as an accurate indicator of autophagic activity [41]. From 24 h post-infection (hpi) onward, the densitometry ratio of the LC3-II to β-actin bands was higher in the lysates of PPRV-infected EECs than in those of mock-infected cells (Figure 1(D)).

To determine whether the elements colocalizing with autophagosomal markers were indeed replicating, we further compared the localization of the LC3 and N proteins in PPRV-infected EECs. As shown in Figure 1(E), PPRV infection resulted in a significant enhancement of LC3 (green) punctate staining signals distributed throughout the entire cytoplasm, whereas the mock-infected EECs exhibited a faint diffuse staining pattern and showed little LC3 punctate accumulation. Moreover, N protein (red) signals in PPRV-infected EECs displayed punctate accumulation, and the N protein red fluorescent punctate staining was highly co-localized with the LC3 green fluorescent punctate staining, which indicates that virus replication is closely associated with the autophagosome-like vesicles. More importantly, 3D images further confirmed this co-localization phenomenon.

Taken together, these results suggest that infection with an attenuated strain of PPRV successfully induces autophagy in host cells.

### PPRV infection enhances autophagic flux

**Figure 2.**
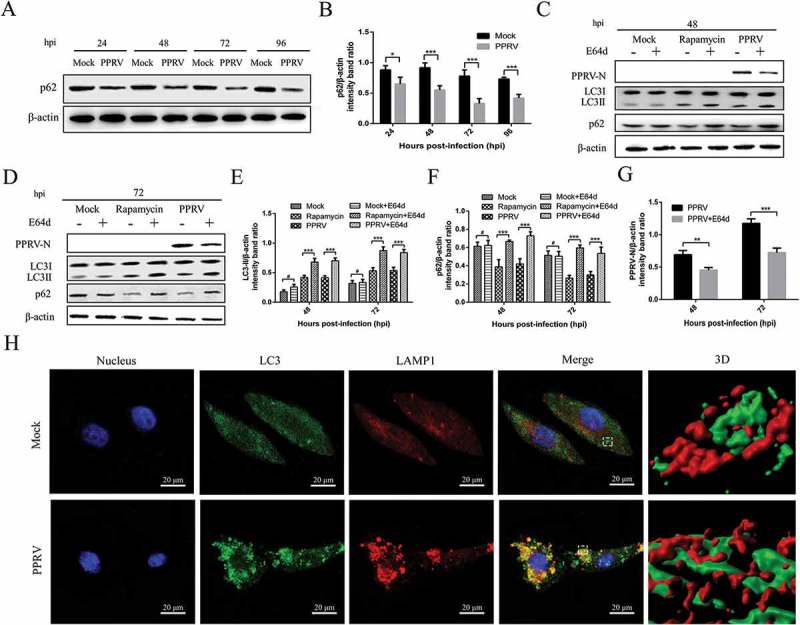
PPRV infection enhances autophagic flux. (a) EECs were mock-infected or infected with PPRV (MOI = 1) for 24, 48, 72 and 96 h. The cell samples were then analysed by immunoblotting with anti-p62 and anti-β-actin (loading control) antibodies. (b) The relative quantification of p62 protein levels compared to β-actin protein levels was determined by densitometry in PPRV-infected cells. (c and d) Cells mock-infected with PBS, cells pre-treated with rapamycin for 12 h and then mock-infected with PBS, and cells infected with PPRV (MOI = 1) were further cultured in the absence and presence of 10 mg/mL E64d for 48 and 72 h. The cell samples were then analysed by immunoblotting with anti-PPRV-N, anti-LC3, anti-p62, and anti-β-actin (loading control) antibodies. (e and f) The relative quantification of LC3 and p62 protein levels compared to β-actin protein levels was determined by densitometry in mock-infected, rapamycin-pre-treated, and PPRV-infected EECs in the absence and presence of E64d. (g) The relative quantification of N protein levels compared to β-actin protein levels was determined by densitometry in PPRV-infected EECs in the absence and presence of E64d. (h) EECs were mock-infected or infected with PPRV (MOI = 1) for 72 h. Then, the cells were fixed and processed for indirect immunofluorescence using antibodies against LC3 and LAMP1 protein. The cell nuclei were counterstained with Hoechst 33342. 3D surface rendered (SR) images in the boxed area are shown in the right panels. Scale bars, 20 μm. The data represent the mean ± SD of three independent experiments. Two-way ANOVA; *P < 0.05; **P < 0.01; ***P < 0.001; ^#^*P *> 0.05.

To determine whether a complete autophagic response was triggered by PPRV infection, we first measured p62 protein degradation. A significant decrease in p62 protein levels was detected in PPRV-infected cells compared to those in mock-infected cells from 24 to 96 hpi (Figure 2(a)). Similarly, the densitometry ratio of the p62 to β-actin bands was much lower in the lysates of PPRV-infected EECs than in those of mock-infected cells (Figure 2(b)).

As shown in Figure 2(c,d), the LC3-II and p62 levels in PPRV-infected cells were significantly increased following E64d treatment at 48 and 72 hpi, further suggesting that the virus indeed enhanced autophagic flux. Similarly, results for LC3-II and p62 expression were also obtained from rapamycin-treated EECs (Figure 2(c,d)). Although the ratio in mock-infected cells was not significantly different, the densitometry ratio of the LC3-II or p62 bands to the β-actin bands was much higher in E64d-treated EECs than in cells not treated with E64d (Figure 2(e,f)). Interestingly, E64d treatment decreased the N protein levels during PPRV infection (Figure 2(c,d,g)). These data indicate that a complete autophagic response can be induced in host cells following PPRV infection.

To further rule out the possibility that autophagosomes could fuse with lysosomes, we investigated the colocalization of LC3 with lysosome-associated membrane protein 1 (LAMP1), a lysosome marker [42]. To this end, both mock- and PPRV-infected EECs were fixed and stained with antibodies against LC3 and LAMP1 and subjected to confocal microscopy at the late time point of 72 hpi. As shown in Figure 2(h), LAMP1 in PPRV-infected EECs was present with a discrete punctate distribution. More importantly, obvious overlapping between the autophagosomes and LAMP1 was also observed in the 3D images of PPRV-infected cells. In contrast, mock-infected cells displayed weak diffuse staining for both LC3 and LAMP1. These data suggested that a portion of the autophagosome fused with the lysosome in PPRV-infected cells. Taken together, our data indicate that PPRV infection significantly enhanced autophagic flux.

### PPRV non-structural and structural protein induces autophagy in EECs

**Figure 3.**
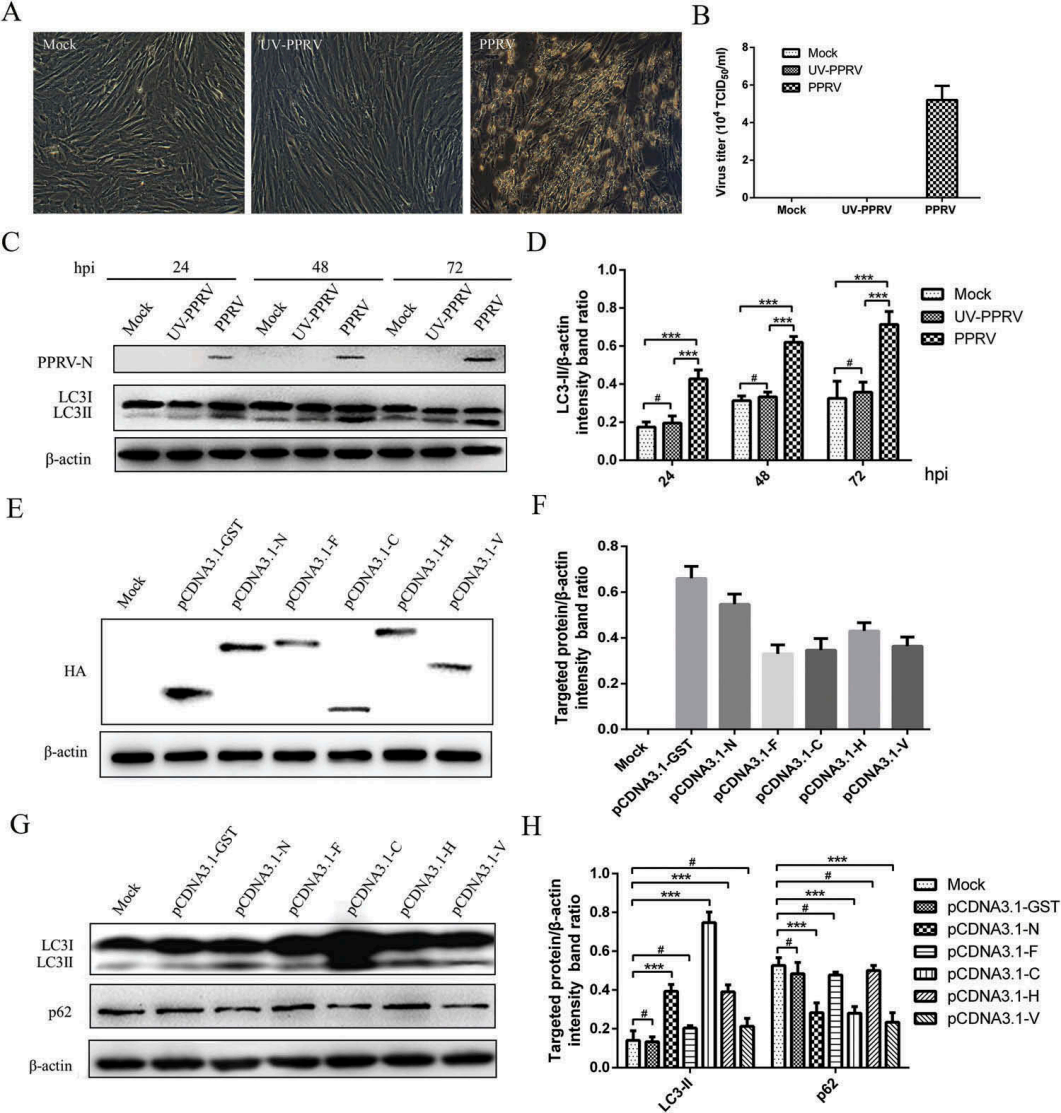
PPRV non-structural and structural proteins induce autophagy in EECs. (a) Morphological changes in mock-, UV-PPRV- and PPRV-infected EECs at 96 h (magnification, × 100). (b) EECs were mock-infected or infected with PPRV (MOI = 1) or UV-inactivated PPRV (MOI = 1) for 96 h. At the end of the infection, virus titres were measured by using the TCID_50_ method. (c) EECs were mock-infected or infected with PPRV or UV-inactivated PPRV (MOI = 1) for 24, 48 and 72 h. The cell samples were then analysed by immunoblotting with anti-PPRV-N, anti-LC3, and anti-β-actin (loading control) antibodies. (d) The relative quantification of LC3 protein levels compared to β-actin protein levels was determined by densitometry in mock-, PPRV- and UV-PPRV-infected cells. (e) EECs were transfected with pCDNA3.1-HA-GST or pCDNA3.1-HA-N, pCDNA3.1-HA-F, pCDNA3.1-HA-C, pCDNA3.1-HA-H and pCDNA3.1-HA-V plasmids expressing the fusion protein for 48 h. The cell samples were then analysed by immunoblotting with an anti-HA antibody. (f) The relative quantification of the viral protein levels compared to the β-actin protein levels was determined by densitometry in transfected cells. (g) EECs were pre-treated as described in (E). The cell samples were then analysed by immunoblotting with anti-LC3, anti-p62, and anti-β-actin (loading control) antibodies. (h) The relative quantification of the target protein levels compared to the β-actin protein levels was determined by densitometry in transfected cells. The data represent the mean ± SD of three independent experiments. Two-way ANOVA; ***P < 0.001; ^#^*P *> 0.05.

To analyse whether PPRV replication was required for the induction of autophagy or whether the uptake of the inactivated virus was sufficient to stimulate autophagosome accumulation, we inactivated PPRV by ultraviolet (UV) radiation and examined its ability to induce autophagy. Active PPRV generated a clear cytopathic effect (CPE) and virus titres in EECs but not in cells inoculated with UV-inactivated PPRV at 96 hpi. These data indicate that UV-inactivated PPRV lost its ability to infect EECs (Figure 3(a,b)). Moreover, compared to the mock-infected cells, the PPRV-infected cells had increased levels of LC3-II from 24 to 72 hpi (Figure 3(c,d)). However, the LC3-II levels in EECs inoculated with UV-inactivated PPRV at the examined time points were similar to those in mock-infected cells. These data indicated that LC3-I in EECs infected by replication-competent PPRV was apparently converted to LC3-II (Figure 3(c,d)), which demonstrated that PPRV replication is necessary for the induction of sustained autophagy. Therefore, PPRV non-structural proteins may also play an important role in the induction of autophagy.

**Figure 4.**
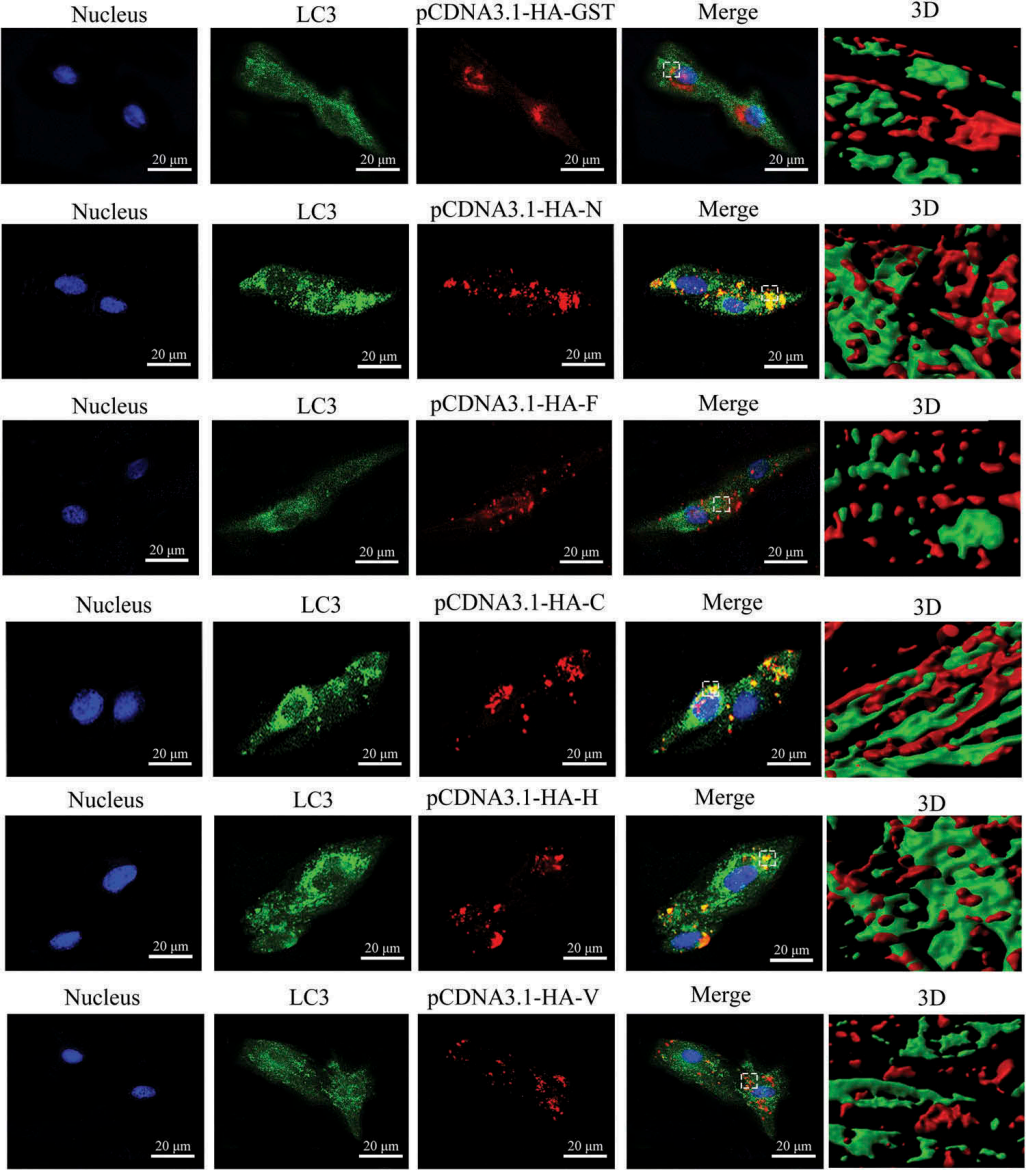
Viral proteins induce the redistribution of LC3 staining signals in EECs. The transfected cells were fixed and processed for indirect immunofluorescence using antibodies against LC3 (green) and HA (red). The cell nuclei were counterstained with Hoechst 33342 solution. 3D surface rendered (SR) images in the boxed area are shown in the right panels. Scale bars, 20 μm.

To further analyse the effect of various viral proteins on autophagy induction during PPRV infection, we expressed N, F, C, H and V as fusion proteins with the HA protein, respectively and transfected the cells with pCDNA3.1-HA-GST as a control. Immunoblotting analyses were used to quantify the expression of HA-GST, HA-N, HA-F, HA-C, HA-H and HA-V. Notably, we detected all of the viral proteins in EECs (Figure 3(e)). Moreover, we found that the expression levels of HA-GST and HA-N were higher than those of other proteins when we transfected the same plasmid concentrations (Figure 3(f)). Interestingly, HA-N, HA-C and HA-H increased the levels of LC3-II, but HA-F and HA-V did not (Figure 3(g,h)). We also found that HA-N, HA-C and HA-V significantly promoted the degradation of p62 in EECs compared with that in pCDNA3.1-HA-GST-transfected and mock-infected cells (Figure 3(g,h)). However, HA-H did not decrease the levels of p62 (Figure 3(g,h)). Consistent with the immunoblotting data, we found that the fluorescence signals and HA-C and HA-H were highly co-localized with the punctate LC3-positive fluorescent staining after transfection with pCDNA3.1-HA-C and pCDNA3.1-HA-H (Figure 4). As shown in Figure 4, the red fluorescent punctate staining of the HA-N protein was also highly co-localized with the green fluorescent punctate staining of LC3. However, a subset of the red fluorescent puncta of HA-GST, HA-F and HA-V did not co-localize with the green fluorescent puncta of LC3 in the 3D images (Figure 4). Collectively, our data reveal the relationship between viral proteins and the autophagic response.

### Induction of autophagy with rapamycin increases virus replication

**Figure 5.**
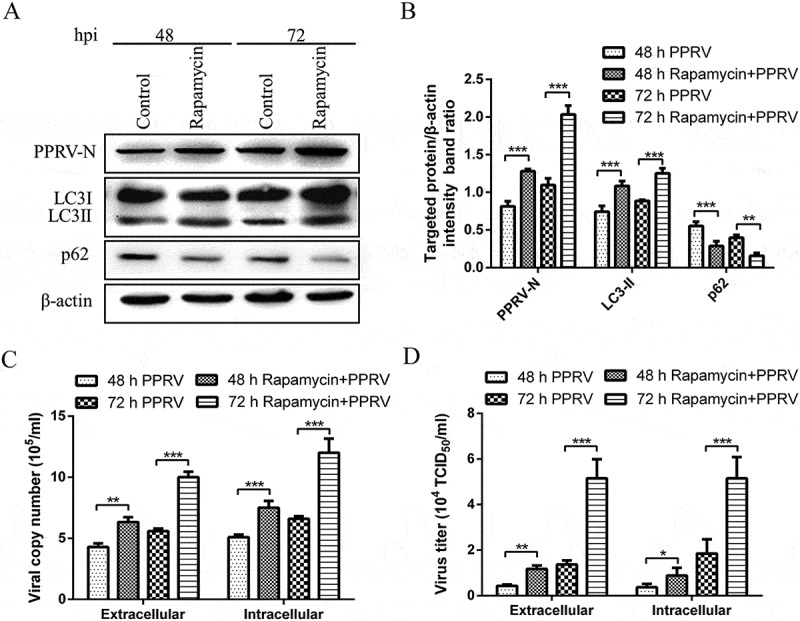
Induction of autophagy with rapamycin increases PPRV replication. (a) EECs were pre-treated with rapamycin for 6 h and then infected with PPRV (MOI = 1) for 48 and 72 h. The cell samples were then analysed by immunoblotting with anti-PPRV-N, anti-LC3, anti-p62, and anti-β-actin (loading control) antibodies. (b) The relative quantification of the target protein levels compared to the β-actin protein levels was determined by densitometry in control and rapamycin-pre-treated cells. (c) EECs were pre-treated and infected as described in (A). At 48 and 72 hpi, both the extracellular and intracellular copy numbers of PPRV were detected by qRT-PCR. (d) EECs were pre-treated and infected as described in (A). At 48 and 72 hpi, both the extracellular and intracellular virus titres were measured by using the TCID_50_ method. The data represent the mean ± SD of three independent experiments. Two-way ANOVA; *P < 0.05; **P < 0.01; ***P < 0.001.

To analyse the role of autophagy in PPRV replication, we examined viral protein N expression and the viral progeny yields following rapamycin treatment. The effect of pharmacological treatment on EEC viability was determined using 3-(4,5-dimethylthiazol-2-yl)-2,5-diphenyl tetrazolium bromide (MTT) assays before formal experiments were conducted, and no significant effect was observed at the selected concentration (Supplementary Figure S1). We found that the induction of autophagy with rapamycin not only upregulated the expression of LC3-II and N in PPRV-infected EECs (Figure 5(a,b)) but also decreased the expression of p62 at 48 and 72 hpi (Figure 5(a,b)). Moreover, a SYBR Green I based real-time RT-PCR assay targeting the P gene of PPRV was established for quantifying viral RNA (Supplementary Figure S2). The correlation between the plasmid dilutions and the threshold cycle (Ct) values according to real-time RT-PCR were analysed by plotting a standard curve (Supplementary Figure S2). A linear regression relationship was observed with a coefficient of determination (R^2^) of 0.9934 and a slope of −3.126 (Supplementary Figure S2). Our data showed that the effect of rapamycin treatment also significantly increased the intracellular and extracellular viral RNA levels (Figure 5(c)). Moreover, the virus titres were consistent with the trends observed in the viral RNA analyses (Figure 5(d)). These findings suggest that the autophagic machinery may not play a defensive role during PPRV infection; rather, it may work to amplify viral RNA synthesis and viral assembly.

### Autophagy inhibition decreases virus replication

**Figure 6.**
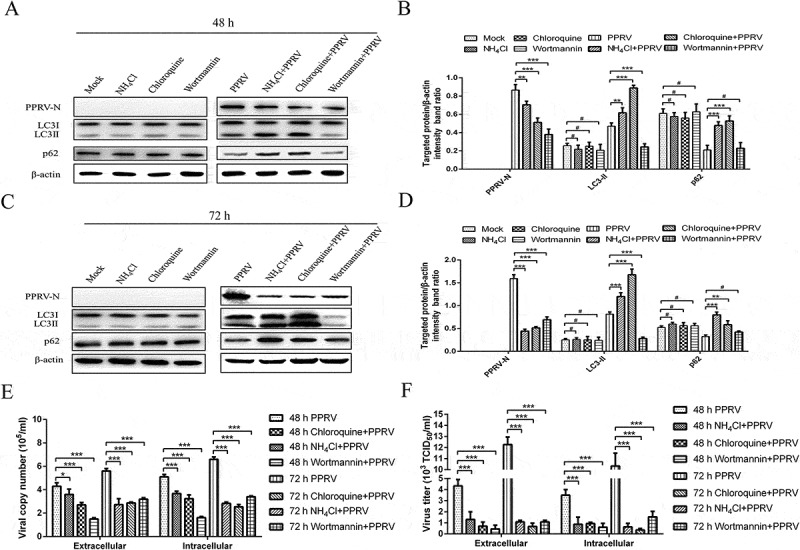
Inhibition of autophagy reduces PPRV replication. (a and c) EECs were pre-treated with NH_4_Cl, chloroquine and wortmannin for 6 h and then infected with PPRV (MOI = 1) for 48 and 72 h. The cell samples were then analysed by immunoblotting with anti-PPRV-N, anti-LC3, anti-p62, and anti-β-actin (loading control) antibodies. (b and d) At 48 and 72 hpi, the relative quantification of the target protein levels compared to the β-actin protein levels was determined by densitometry in control and NH_4_Cl-, chloroquine- and wortmannin-pre-treated cells. (e) EECs were pre-treated and infected as described in (A and C). At 48 and 72 hpi, both the extracellular and intracellular copy numbers of PPRV were detected by qRT-PCR. (f) EECs were pre-treated and infected as described in (A and C). At 48 and 72 hpi, both the extracellular and intracellular virus titres were measured by using the TCID_50_ method. The data represent the mean ± SD of three independent experiments. Two-way ANOVA; *P < 0.05; **P < 0.01; ***P < 0.001; ^#^*P *> 0.05.

To further determine the effect of autophagy on PPRV replication, we exposed EECs to NH_4_Cl, chloroquine and wortmannin and analysed PPRV replication by detecting viral protein N expression and viral progeny yields by using the relevant assays. As shown in Figure 6(a-d), NH_4_Cl, chloroquine and wortmannin treatment did not significantly influence the expression of LC3-II and p62 at 48 and 72 h in mock-infected EECs. However, NH_4_Cl and chloroquine treatment increased the expression of LC3-II and p62 at 48 and 72 hpi in PPRV-infected EECs, and wortmannin treatment reduced the expression of LC3-II at 48 and 72 hpi in PPRV-infected EECs (Figure 6(a-d)). Importantly, we found that the expression level of PPRV-N was significantly decreased following NH_4_Cl, chloroquine and wortmannin treatment in PPRV-infected EECs (Figure 6(a-d)). Similarly, the effects of NH_4_Cl, chloroquine and wortmannin treatment on the extracellular and intracellular viral RNA levels and titres were also significantly decreased (Figure 6(e,f)). These data indicated that the different stages of autophagy are very closely related to virus replication.

**Figure 7.**
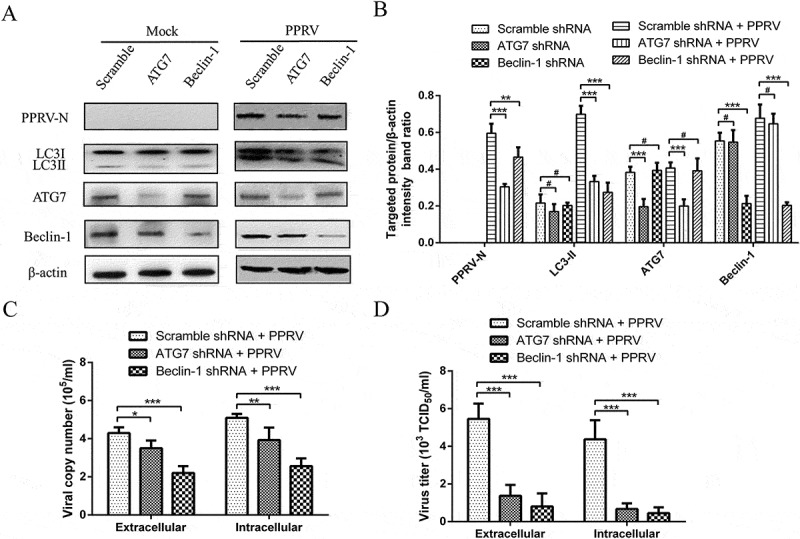
Inhibition of autophagy with specific shRNAs targeting Beclin-1 and ATG7 reduces PPRV replication. (a) EECs were transfected with shRNAs targeting Beclin-1 or ATG7 or scrambled shRNAs for 48 h; then, the cells were infected with PPRV (MOI = 1) for 48 h. The cell samples were then analysed by immunoblotting with anti-PPRV-N, anti-LC3, anti-ATG7, anti-Beclin-1, and anti-β-actin (loading control) antibodies. (b) The relative quantification of the target protein levels compared to the β-actin protein levels was determined by densitometry in transfected shRNA cells. (c) EECs were pre-treated and infected as described in (A). At 48 hpi, both the extracellular and intracellular copy numbers of PPRV were detected by qRT-PCR. (d) EECs were pre-treated and infected as described in (A). At 48 hpi, both the extracellular and intracellular virus titres were measured by using the TCID_50_ method. The data represent the mean ± SD of three independent experiments. Two-way ANOVA; *P < 0.05; **P < 0.01; ***P < 0.001; ^#^*P *> 0.05.

To extend the studies with pharmacological regulators, we examined the effects of depleting the expression of the endogenous autophagy proteins ATG7 and Beclin-1 on PPRV replication using target-specific RNA interference. As shown in Figure 7(a,b), mock- and PPRV-infected EECs transfected with shATG7 and shBeclin-1 presented with significantly decreased protein levels of endogenous ATG7 and Beclin-1 compared with scrambled shRNA-transfected cells, indicating that the expression of the target proteins was successfully inhibited in the EECs. Importantly, the decreases in the levels of ATG7 and Beclin-1 were also reflected in a significant decrease in the amount of LC3-II compared with the scrambled shRNA group in PPRV-infected cells (Figure 7(a,b)). Moreover, the suppression of ATG7 and Beclin-1 expression strongly reduced the expression of the viral N protein (Figure 7(a,b)) and the viral progeny yields (Figure 7(c,d)) in PPRV-infected EECs. These data further reveal that autophagy plays an important role in the replication of PPRV in host cells.

### Inhibition of autophagosome formation accelerates apoptosis in PPRV-infected EECs

**Figure 8.**
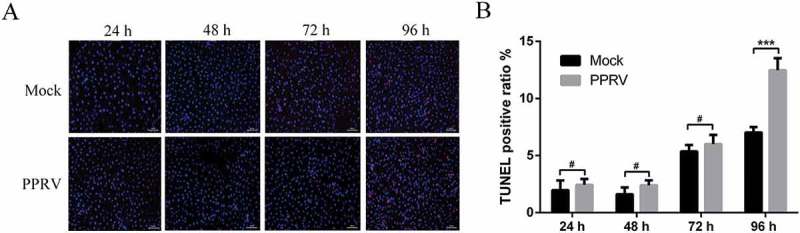
TUNEL labelling of PPRV-infected EECs. (a) EECs were mock-infected or infected with PPRV (MOI = 1) for 24, 48, 72 and 96 h. The cell samples were labelled with TUNEL and then counterstained with Hoechst 33342 solution. Scale bars, 100 μm. (b) Percentage of TUNEL-positive mock-infected and PPRV-infected EECs at the indicated time points. The data represent the mean ± SD of three independent experiments. Two-way ANOVA; ***P < 0.001; ^#^*P *> 0.05.

We have indicated that PPRV-infected EECs displayed autophagy induction (Figure 1). In addition to the autophagic pathway, other forms of cell survival stress, such as apoptosis pathways, may be induced in cells subjected to viral infection. These results prompted us to investigate whether apoptosis pathways are associated with PPRV in EECs. As shown in Figure 8(a,b), TUNEL-labelling was not different between virus-infected and mock-infected cells within 72 hpi. Interestingly, more TUNEL-positive cells were observed in PPRV-infected groups than in mock-infected groups at 96 hpi. These data demonstrate that apoptosis may play an important role following PPRV infection in host cells.

**Figure 9.**
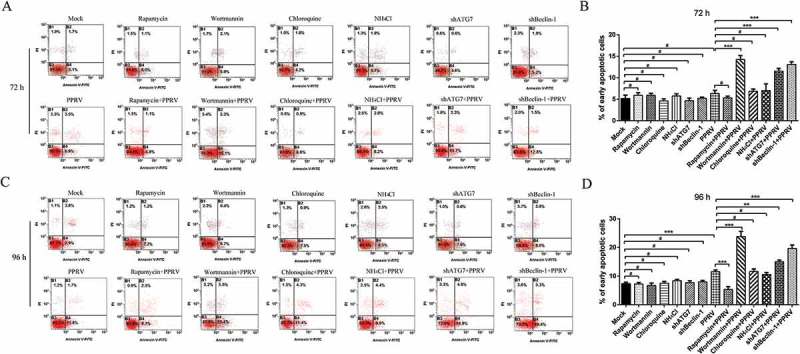
Inhibiting autophagosome formation accelerates apoptosis in EECs with PPRV infection. (a) EECs were pre-treated with rapamycin, wortmannin, NH_4_Cl, chloroquine, and shRNAs targeting Beclin-1 and ATG7 and then infected with PPRV (MOI = 1) for 72 h. The cell samples were dual-labelled with Annexin V and PI and analysed by flow cytometry. (b) Proportions of apoptotic cells in panel A. (c) EECs were pre-treated and infected with PPRV (MOI = 1) for 96 h. The cell samples were analysed by flow cytometry. (d) Proportions of apoptotic cells in panel C. The data represent the mean ± SD of three independent experiments. Two-way ANOVA; **P < 0.01; ***P < 0.001; ^#^*P *> 0.05.

To further verify the relationship between the autophagy and apoptosis induced by PPRV in the different stages of autophagy, the pre-treated and infected cells were then collected and subjected to flow cytometry analysis with fluorescein isothiocyanate (FITC)-Annexin V and propidium iodide (PI). In agreement with the TUNEL assay results, no obvious signs of apoptosis were observed in either mock- or PPRV-infected cells at 72 hpi (Figure 9(a,b)). However, PPRV-infected cells presented with a significant level of apoptosis compared to mock-infected cells at 96 hpi (Figure 9(c,d)). As shown in Figure 9(a-d), rapamycin, NH_4_Cl, chloroquine, wortmannin, ATG shRNA and Beclin-1 shRNA treatment did not significantly influence the number of apoptotic cells at 72 and 96 h in mock-infected EECs, indicating that the chemical autophagy inhibitor, activator and shRNA cannot regulate EEC apoptosis in the absence of PPRV. Interestingly, rapamycin treatment significantly decreased the number of apoptotic cells at 96 hpi in PPRV-infected EECs (Figure 9(c,d)). Moreover, NH_4_Cl and chloroquine treatment did not cause significant changes in the number of apoptotic cells at 72 and 96 hpi, indicating that inhibiting the fusion of autophagosomes and lysosomes did not promote apoptosis in PPRV-infected EECs. Importantly, the inhibition of autophagy with wortmannin significantly increased the number of apoptotic cells at 72 and 96 hpi in PPRV-infected EECs (Figure 9(a-d)). With PPRV infection and the RNA interference approach at 72 and 96 hpi, the knockdown of ATG7 and Beclin-1 remarkably increased the number of apoptotic cells (Figure 9(a-d)), suggesting that the inhibition of autophagosome formation strengthened the cell apoptosis induced by PPRV. Therefore, our data reveal that autophagy and apoptosis are two important processes that interact with each other during PPRV infection.

### Autophagosome formation inhibition induced caspase-dependent apoptosis in PPRV-infected EECs

**Figure 10.**
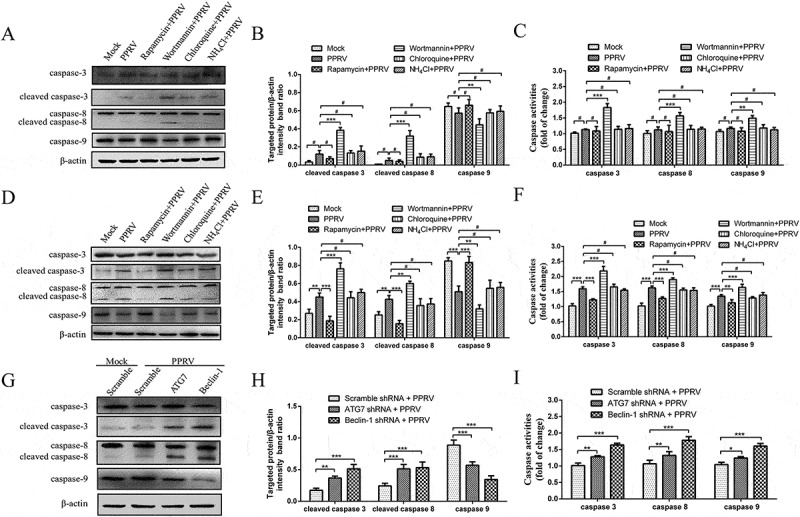
Inhibiting autophagosome formation induced caspase-dependent apoptosis in EECs with PPRV infection. (a and d) EECs were mock-infected and treated with rapamycin, NH_4_Cl, chloroquine or wortmannin, followed by PPRV infection (MOI = 1) for 72 (A) and 96 h (D). The cell samples were then analysed by immunoblotting with anti-caspase-3, anti-caspase-8, anti-caspase-9, and anti-β-actin (loading control) antibodies. (b and e) At 72 (B) and 96 hpi (E), the relative quantification of the target protein levels compared to the β-actin protein levels was determined by densitometry in treated cells. (c and f) EECs were pre-treated and infected as described in (A and D). At 72 (C) and 96 hpi (F), the activities of caspase-3, caspase-8 and caspase-9 were detected using colorimetric assay kits. (g) EECs were transfected with shRNAs targeting Beclin-1 or ATG7 or scrambled shRNAs for 48 h and then infected with PPRV (MOI = 1) for 72 h. The cell samples were then analysed by immunoblotting with anti-caspase-3, anti-caspase-8, anti-caspase-9, and anti-β-actin (loading control) antibodies. (h) The relative quantification of the target protein levels compared to the β-actin protein levels was determined by densitometry in transfected shRNA cells. (i) EECs were pre-treated and infected as described in (G). The activities of caspase-3, caspase-8 and caspase-9 were detected using colorimetric assay kits. The data represent the mean ± SD of three independent experiments. Two-way ANOVA; *P < 0.05; **P < 0.01; ***P < 0.001; ^#^*P *> 0.05.

The apoptosis pathway is regulated by the actions of a series of caspases [27]. Caspase-3, caspase-8, and caspase-9 were significantly activated in PPRV-infected EECs compared with mock-infected EECs at 96 hpi but not at 72 hpi (Figure 10(a-f)). In agreement with the flow cytometry analyses, our data showed that rapamycin, NH_4_Cl, chloroquine, wortmannin, ATG shRNA and Beclin-1 shRNA treatment did not significantly influence the activities of caspase-3, caspase-8 and caspase-9 in mock-infected EECs (Supplementary Figure S3). To further determine the relationship between autophagy and apoptosis in PPRV-infected EECs, the cleavage of caspase-3, caspase-8 and caspase-9 and caspase activity were analysed after the infected cells were pre-treated with rapamycin, NH_4_Cl, chloroquine, wortmannin, ATG7 shRNA or Beclin-1 shRNA. As shown in Figure 10(d-f), the induction of autophagy with rapamycin inhibited the activities and the cleavage of caspase-3, caspase-8, and caspase-9 compared with the absence of rapamycin treatment in PPRV-infected EECs at 96 hpi. However, the inhibition of autophagy with NH_4_Cl or chloroquine did not affect the activities or the cleavage of caspase-3, caspase-8, and caspase-9 at the examined time points in PPRV-infected cells (Figure 10(a-f)). Interestingly, the inhibition of autophagy with wortmannin promoted the activities and cleavage of caspase-3, caspase-8, and caspase-9 at 72 and 96 hpi following PPRV infection (Figure 10(a-f)). Furthermore, our results showed that both cell lines transfected with ATG7 and Beclin-1 shRNA presented with significantly increased activity levels and cleavage of caspase-3, caspase-8, and caspase-9 compared with cells transfected with scrambled shRNA during PPRV infection for 72 h (Figure 10(g-i)). These results indicate that PPRV-induced autophagosome formation limits apoptosis by inhibiting caspase-dependent apoptosis in host cells.

## Discussion

Like all morbilliviruses, PPRV has an established lymphatic and epithelial tropism [43]. Among the cells subject to PPRV infection, Vero cells are usually used to study PPRV infection in vitro [17,44]. It is well known that PPRV can infect a multitude of host cells, such as sheep kidney epithelial cells [45] (PO cell line) and goat peripheral blood mononuclear cells (PBMCs) [46]. Throughout our study, caprine endometrial epithelial cells were used for the experiments, and we showed that PPRV can successfully replicate in caprine EECs, further confirming the clinical phenomenon of abortions in PPRV-infected goats [3,4]; these data thus provide a theoretical basis for the replication mechanism of PPRV. Currently, increasing evidence suggests that several genetic connections participate in crosstalk between autophagy and apoptosis [47]. Although previous studies have demonstrated that autophagy and apoptosis are both induced by PPRV infection [17,36], it is not yet known whether crosstalk might exist between autophagic and apoptotic pathways in host cells subjected to PPRV infection. In this study, we used a PPRV vaccine strain (Nigeria 75/1) to investigate the role of autophagy in viral replication and the relationship between autophagy and apoptosis in EECs.

Autophagy plays an important role in pathogenic infection, which is involved in the mechanism of balance between stimuli and cells. In recent years, an increasing number of studies have demonstrated that the infection processes of morbilliviruses are closely related with autophagic flux [16,17]. However, the coxsackievirus B3, herpes simplex virus and influenza A virus have been shown to induce autophagosome formation but block the fusion of autophagosomes with lysosomes [48–50]. More importantly, MeV infection induces successive autophagic signalling, leading to a sustained increase in autophagic flux [16]. Our data are consistent with those of MeV; indeed, E64d treatment increased the amount of LC3-II in both rapamycin-treated and virus-infected EECs, indicating that there was increased autophagic flux. Immunoblotting analyses for changes in the protein levels of p62 further verified that autophagic flux increased upon PPRV infection. Furthermore, LAMP1 is also required for the fusion of autophagosomes and endosomal vesicles [51]. Our data showed that endogenous LC3 partially overlapped with LAMP1 in PPRV-infected EECs. It is suggested that PPRV infection induced the fusion of autophagosomes with acidified LAMP1-positive lysosomes and thereby strengthened the degradation of autophagy substrates. Since autophagy was successfully induced in host epithelial cells, we conclude that autophagy is a necessary outcome of PPRV infection in cell cultures.

Several reports showed that autophagy is activated upon viral replication [19,32]. Our results show that UV-inactivated PPRV lost its ability to induce autophagy, indicating that viral replication was required for PPRV infection-induced autophagy. In contrast, low basal levels of LC3-II were present in mock-infected cells, indicating that basal autophagy in normal EECs was essential for maintaining homeostasis. Recent studies show that several members of the *Paramyxoviridae* family, including MeV [16], human respiratory syncytial virus [52], canine distemper virus [53] and Newcastle disease virus [35], can utilize an autophagy mechanism to facilitate replication. To further determine what role autophagy plays in the replication of PPRV, we examined viral replication ability by exposing EECs to rapamycin, an inducer of autophagy that specifically inhibits the action of mTOR [54]. Our data showed that rapamycin treatment not only upregulated the expression of the viral N protein but also increased the yield of PPRV progeny. Moreover, the inhibition of autophagy in the replication of PPRV still needs to be examined in depth. NH_4_Cl and chloroquine inhibit autophagy by reducing the acidity of lysosomes and inhibiting the fusion of autophagosomes and lysosomes [55]. We found that NH_4_Cl and chloroquine treatment significantly increased the accumulation of LC3-II and p62 following PPRV infection. The increase in the levels of both LC3-II and p62 in PPRV-infected cells after NH_4_Cl and chloroquine treatment was only a portion of what had been turned over by lysosomal proteolysis. Wortmannin inhibits autophagy by blocking the formation of autophagosomes [56], which could not increase the accumulation of LC3-II and p62 following PPRV infection. Importantly, both the chemical inhibitor and shRNA depletion of the essential autophagy proteins ATG7 and Beclin-1 downregulated N expression and decreased the yield of PPRV progeny. Based on these results, we conclude that the different stages of the autophagy pathway serve a valuable role in PPRV infection, rather than blocking the virus spread in host cells. It is important to note that the effect of the mutative levels of autophagy on extracellular viral titres and RNA levels were the same as those on intracellular viral titres and RNA levels, indicating that autophagy is a regulator of the release of cytoplasmic viruses.

The role of the C protein of morbilliviruses has been suggested to regulate efficient virus replication in host cells, virulence determination, and RdRp activity by interacting with the host cell protein SHCBP1 and blocking the induction of type I interferons [57–59]. One recent observation is that the expression of MeV-C is required for the induction of late autophagy in infected HeLa cells, subsequent to MeV replication [16]. In the present study, we found that PPRV-C significantly increased the level of LC3-II and promoted the degradation of p62. These results indicated that PPRV-C can induce autophagosome formation and activate a complete autophagic response. MeV can exploit autophagy through the physical interaction of the MeV-C protein with the autophagy-associated protein IRGM (immunity-related GTPase M) [60]. Moreover, the C protein has a high degree of homology with the morbilliviruses [61]. The molecular mechanism for how PPRV-C affects the autophagy process related to IRGM needs to be examined in greater detail. Unlike the C protein, the V protein is phosphorylated and can bind both the N and L proteins; therefore, it is believed to regulate RNA synthesis [62]. Our data clearly demonstrate that the PPRV-V protein did not modulate autophagosome formation but significantly decreased the degradation of p62. It is likely that the V protein is important and necessary for the induction of autophagic flux by PPRV. Interestingly, we also found that PPRV-N increased the level of LC3-II and promoted the degradation of p62. The contribution of the different autophagy signals in PPRV maturation remains to be investigated. Moreover, autophagy induction has been reported after co-expression of the F and H proteins in different members of the *Paramyxoviridae* family, strongly indicating that there is a common link between membrane fusion and autophagy [63]. Our data showed that PPRV-H in the cytoplasm of EECs significantly increased the level of LC3-II. Moreover, PPRV-H in the cytoplasm of EECs cannot promote the degradation of p62, indicating that the intracellular H protein activates an incomplete autophagic response in host epithelial cells. In addition, MeV induces autophagy through the CD46-Cyt-1/GOPC pathway, which indicates that a cell surface pathogen receptor can directly trigger autophagy, a critical step for controlling infection [41,64]. Recent reports also demonstrate that SLAM recruits the vps34/Beclin-1 autophagic complex, suggesting that morbilliviruses might induce autophagy pathways upon receptor binding with the H protein [65]. Importantly, nectin-4 was identified as the epithelial receptor for several strains of MeV and PPRV [45,66]. To reinforce the theory that receptors could induce autophagy upon pathogen binding, autophagy induction mediated by nectin-4 and PPRV-H in host epithelial cells still needs to be examined in detail. In addition, our confocal immunofluorescence analyses demonstrated that the PPRV structural proteins N and H colocalized with LC3 in host cells. However, colocalization between GFP-LC3 and the MeV-N protein was not observed [16]. These results suggest that significant differences between the replication mechanisms and characteristics exist between different viruses of the same family, which may be related to host specificity, cell types and different pathogenic mechanisms of the infections. Interestingly, the non-structural protein C also colocalized with LC3 in host cells. These results indicated that the PPRV-C protein may utilize the membranes of autophagosome-like vesicles to enhance its effects in regulating intracellular responses. Undoubtedly, both of the experiments indicate that the replication complexes of PPRV were formed on the membranes of autophagosome-like vesicles. However, we did not investigate whether analogues of the LC3-I-positive vesicular structures (termed EDEMosomes), which are hijacked by viruses for their replication [67], exist within the autophagosome-like vesicles induced by PPRV infection. Further investigation is needed to conclusively answer this question.

The recent study has shown the importance of the crosstalk between autophagy and apoptosis in virus infection. For example, DRAM (damage-regulated autophagy modulator), a p53 target gene encoding a lysosomal protein, also links autophagy to p53 and programmed cell apoptosis [68]. The MeV-V protein interacts with the DNA binding domains of p53 family proteins to inhibit virus-induced cell apoptosis [69]. Indeed, the inhibition of apoptosis strongly facilitates MeV replication [63]. Furthermore, the inhibition of autophagy causes a growth defect and induces more apoptosis in infected cells, suggesting that the main function of autophagy in the course of MeV infection is to delay MeV-induced apoptosis [16]. In this study, we found that the inhibition of autophagy at different stages reduces viral particle release, which was achieved through the effects on the apoptosis. Interestingly, the inhibition of autophagosome formation with wortmannin induced caspase-dependent apoptosis in EECs in PPRV infection. However, in the fusion stage of autophagosomes and lysosomes, the inhibition of autophagy with NH_4_Cl and chloroquine did not increase the number of apoptotic cells. These data indicated that autophagosome formation plays a critical anti-apoptosis role during PPRV infection. Moreover, the expression products of the ATG7 and Beclin-1 genes are essential for the activation of autophagosome formation, and knocking down the ATG7 and Beclin-1 genes can impair the autophagic pathway [70,71]. Interestingly, Beclin-1 is required for ATG7-dependent and ATG7-independent autophagy [72]. Beclin-1 also regulates apoptosis by interacting with both BCL2 and BCL2L1/BCL-XL [73]. Additionally, apoptosis may inhibit autophagy as CAPN/calpain cleaves ATG5, which triggers CASP-dependent cell death [71,74]. Caspases can also cleave Beclin-1 in apoptosis, thereby destroying its pro-autophagic activity [75]. In this study, caspase-3, caspase-8 and caspase-9 mediated pathway activation was triggered by PPRV in both ATG7 and Beclin-1 knockdown cells. These results suggested that Beclin-1 and ATG7 may facilitate virus replication via regulating autophagy and apoptosis in PPRV-infected EECs.

In conclusion, our study reveals a novel approach to improve the production efficiency of live attenuated PPRV vaccines, which may enhance their immune effect in host cells by targeting the interplay between the autophagy and apoptosis pathways. Further investigating the immunological function of autophagy as well as its role in the immune escape of PPRV might be of importance for controlling viral infection and the better usage of PPRV-derived vaccines for therapy.
