# Supplementary figures and images for: Autophagy enhances the replication of Peste des petits ruminants virus and inhibits caspase-dependent apoptosis in vitro
Source: Virulence. 2018 Aug 1;9(1):1176–94. doi: 10.1080/21505594.2018.1496776 (PMC6086290; doi:10.1080/21505594.2018.1496776)

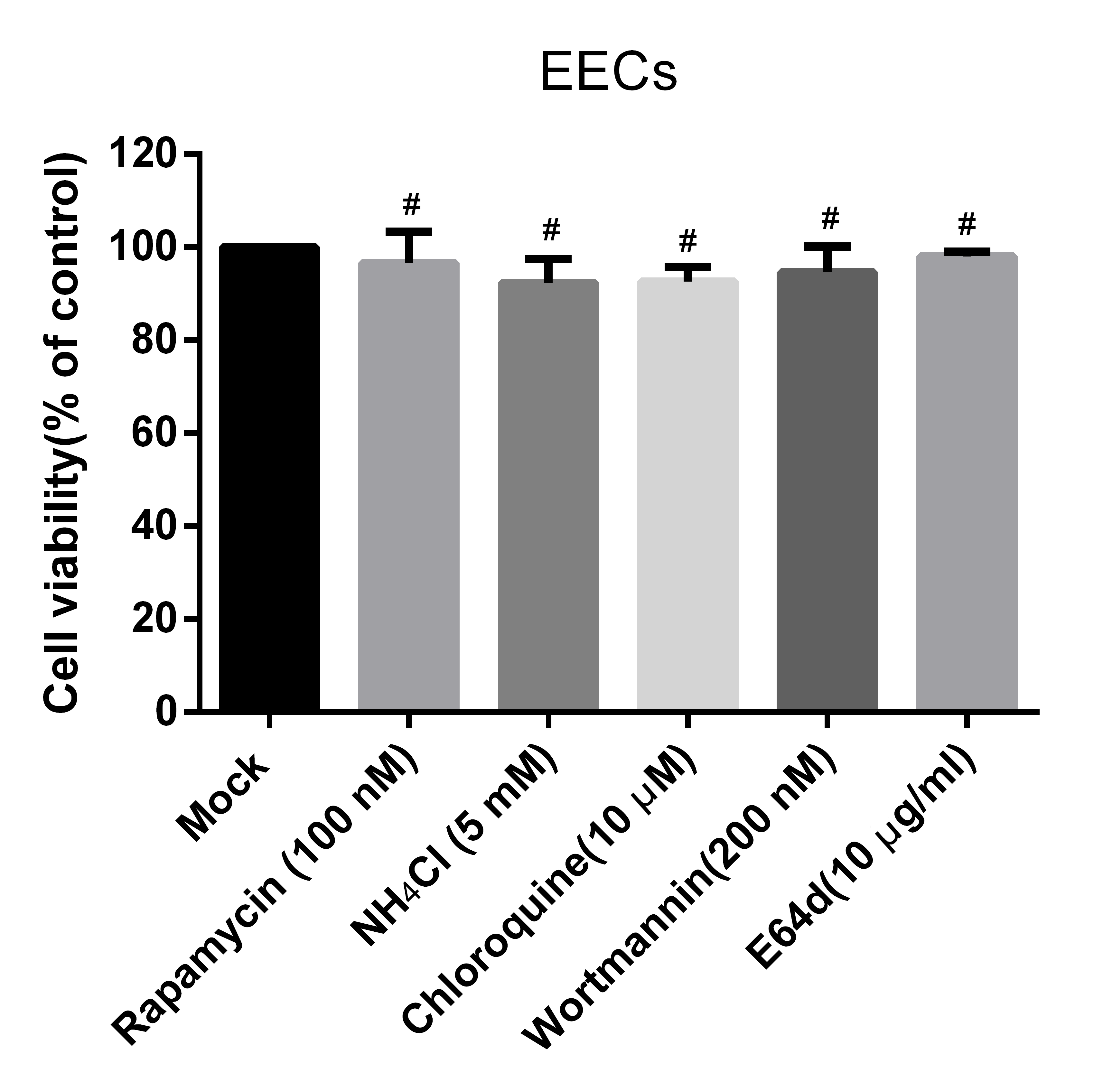

Supplement: Supplemental Material [file kvir-09-01-1496776-s001.zip › Supplementary Fig.S1.tif]

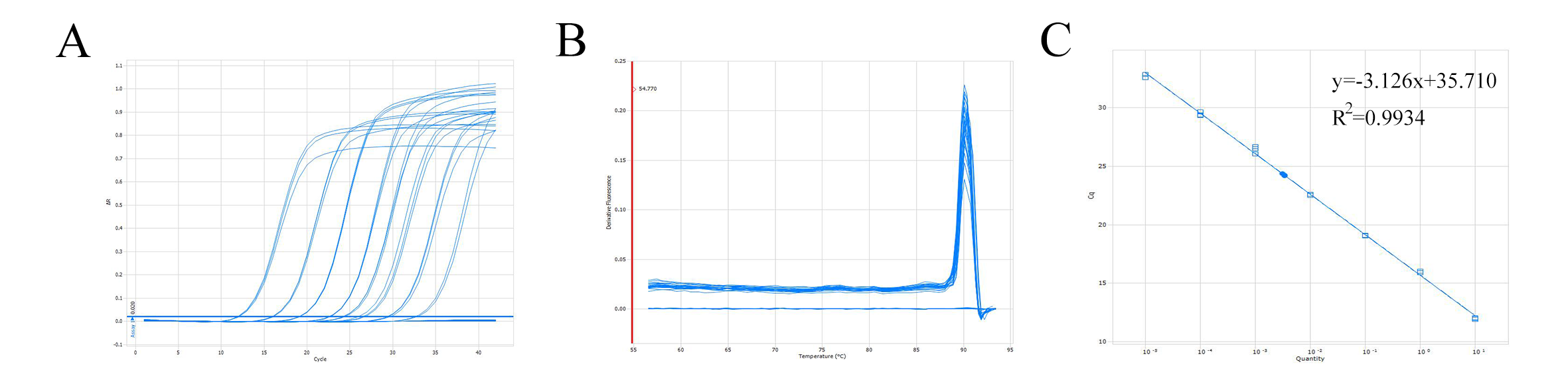

Supplement: Supplemental Material [file kvir-09-01-1496776-s001.zip › Supplementary Fig.S2.tif]

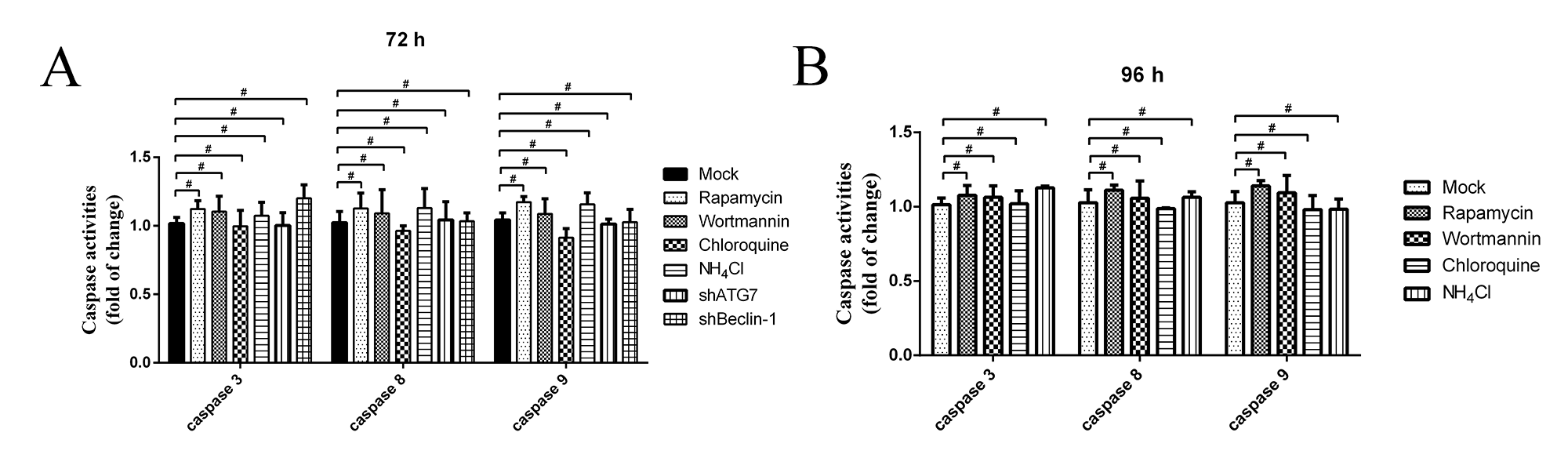

Supplement: Supplemental Material [file kvir-09-01-1496776-s001.zip › Supplementary Fig.S3.tif]
